# A catalogue of somatic NRF2 gain-of-function mutations in cancer

**DOI:** 10.1038/s41598-018-31281-0

**Published:** 2018-08-27

**Authors:** Michael John Kerins, Aikseng Ooi

**Affiliations:** Department of Pharmacology and Toxicology, College of Pharmacy, University of Arizona, Tucson, AZ 85721 USA

## Abstract

Identification and characterization of somatic mutations in cancer have important prognostication and treatment implications. Genes encoding the Nuclear factor (erythroid-derived 2)-like 2 (NRF2) transcription factor and its negative regulator, Kelch-like ECH-associated protein 1 (KEAP1), are frequently mutated in cancer. These mutations drive constitutive NRF2 activation and correlate with poor prognosis. Despite its apparent significance, a comprehensive catalogue of somatic NRF2 mutations across different tumor types is still lacking. Here, we catalogue NRF2 mutations in The Cancer Genome Atlas (TCGA) database. 226 unique NRF2-mutant tumors were identified from 10,364 cases. NRF2 mutations were found in 21 out of the 33 tumor types. A total of 11 hotspots were identified. Of these, mutation to the R34 position was most frequent. Notably, R34 and D29 mutations were overrepresented in bladder, lung, and uterine cancers. Analyses of corresponding RNA sequencing data using a de novo derived gene expression classifier showed that the R34 mutations drive constitutive NRF2 activation with a selection pressure biased against the formation of R34L. Of all R34 mutants, R34L conferred the least degree of protein stabilization, suggesting a pro-tumor NRF2 half-life threshold. Our findings offer a comprehensive catalogue of NRF2 mutations in cancer that can help prognostication and NRF2 research.

## Introduction

The NRF2 (Nuclear factor (erythroid-derived 2)-like 2) transcription factor is the master regulator of cellular antioxidant responses^1^. When activated, NRF2 promotes the transcription of its target genes, many of which are involved in the augmentation of cellular reducing capacity and in the detoxification and efflux of xenobiotics^2–4^. Thus, NRF2 activation protects cells against chemical and oxidative insults.

Several studies have shown that *NRF2*, together with its negative regulator *KEAP1* (Kelch-like ECH-associated protein 1), are frequently mutated in cancer^5–11^. These mutations phenotypically converged at the constitutive activation of NRF2^12,13^. Since NRF2 activation protects cells against xenobiotics and oxidative insults, these mutations correlate with chemo- and radio-resistance, and result in poor clinical outcomes^13^. Indeed, patient survival has been shown to be significantly poorer in tumors harboring NRF2 activation, including lung^14,15^, gallbladder^16^, esophageal^17^, ovarian^18^, head and neck^19^, and gastric cancers^20^. As such, identification of cancer cases with constitutive NRF2 activation has important prognostic implication.

Mechanistic studies have revealed that KEAP1 interacts with NRF2 through the conserved ETGE and DLG motifs residing in the N-terminal tail of NRF2^21^. These interactions enable KEAP1 to function as a substrate adaptor protein for a Cullin-3 (CUL3) containing E3 ubiquitin ligase complex that mediates NRF2 ubiquitylation^22^. In this system, KEAP1 functions as a cellular redox sensor, whereby critical cysteine residues on its surface are amenable to covalent modification by electrophiles and reactive oxygen species^23–25^. These modifications render KEAP1 unable to mediate NRF2 ubiquitylation, enabling NRF2 to accumulate and perform its transcriptional function. Accordingly, somatic *NRF2* mutations in cancer mainly occurred within the ETGE and DLG motifs^8^. The focal nature of somatic *NRF2* mutations presents an attractive genetic screening modality that could be used to identify cancers with constitutive NRF2 activation. Moreover, the mutant forms of NRF2 are unique to cancer cells and therefore may be targeted as a treatment strategy. However, despite the focal nature of *NRF2* mutation, a thorough cataloging of *NRF2* mutation in cancer has yet to be reported, hampering the development of an easy *NRF2* mutation screening method.

Here we catalogue somatic *NRF2* mutations in cancer cases reported in The Cancer Genome Atlas (TCGA). By cross analyzing somatic mutations with gene expression analyses, we report *NRF2* mutations that result in constitutive NRF2 activation.

## Results

### Consensus somatic mutation calling in TCGA data

**Figure 1:**
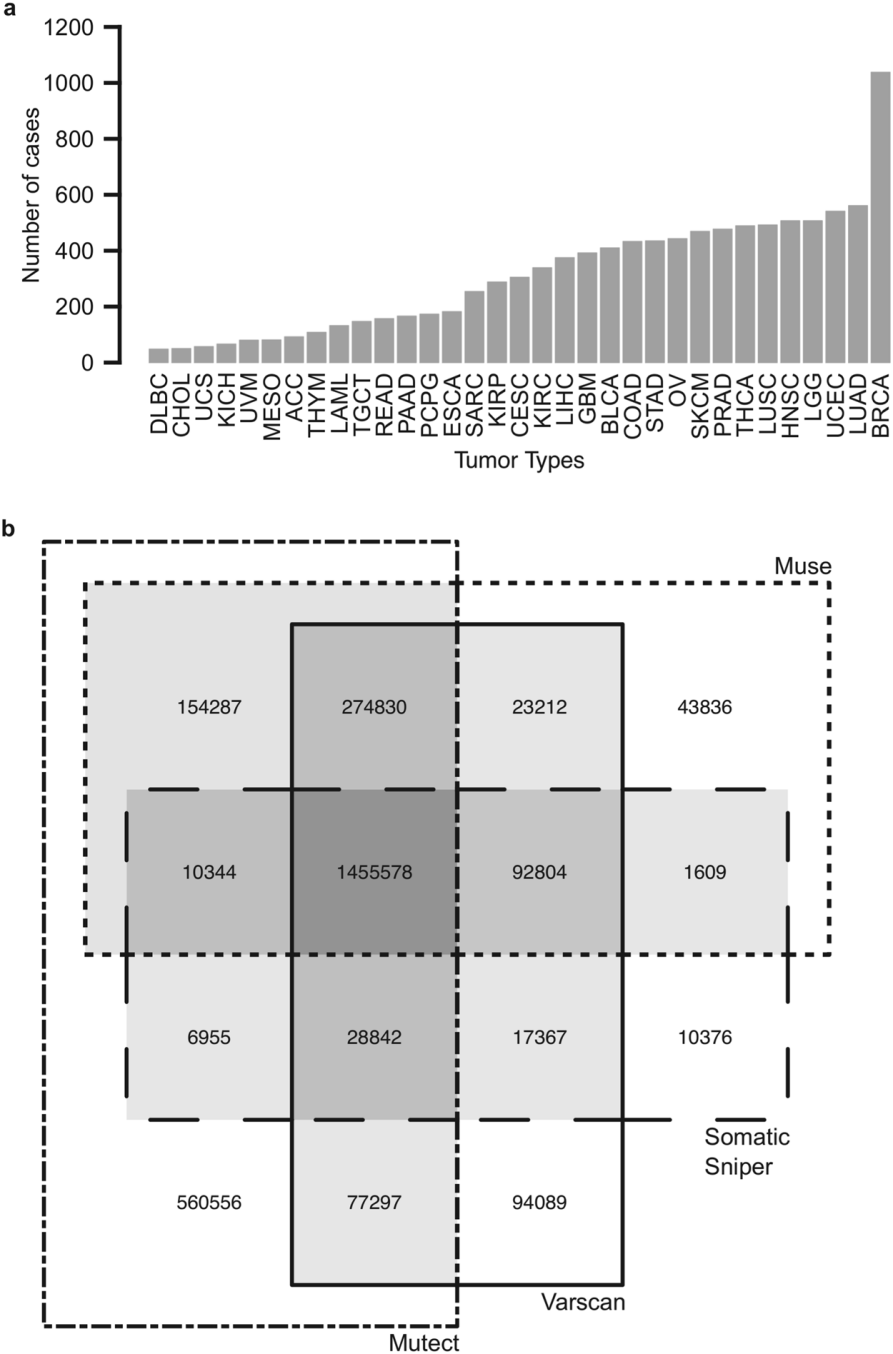
Overview of TCGA dataset. (**a**) Number of cases analyzed within each tumor type. (**b**) Overlaps of unique mutations identified by different mutation calling algorithms. Mutations identified by two or more algorithms (shaded) were used in downstream analyses.

We obtained somatic mutation data for 10,364 tumor cases, spanning across 33 different tumor types (Fig. 1a). TCGA utilizes 4 different somatic mutation-calling algorithms, which have slight differences in sensitivity and specificity^26,27^. For the purpose of cataloging somatic *NRF2* mutations, we used mutation sites that were called by at least two of the 4 mutation calling algorithms to achieve a compromise between mutation calling accuracy and sensitivity. This restricted the total mutation calls from 2,851,982 down to 2,143,125 unique mutation sites (Fig. 1b). Importantly, all *NRF2* and *KEAP1* mutations were concurrently called by all 4 somatic mutations calling algorithms, indicating the high confidence of those calls.

### NRF2 and KEAP1 mutations are overrepresented in tumors with known association to carcinogen exposure

**Figure 2:**
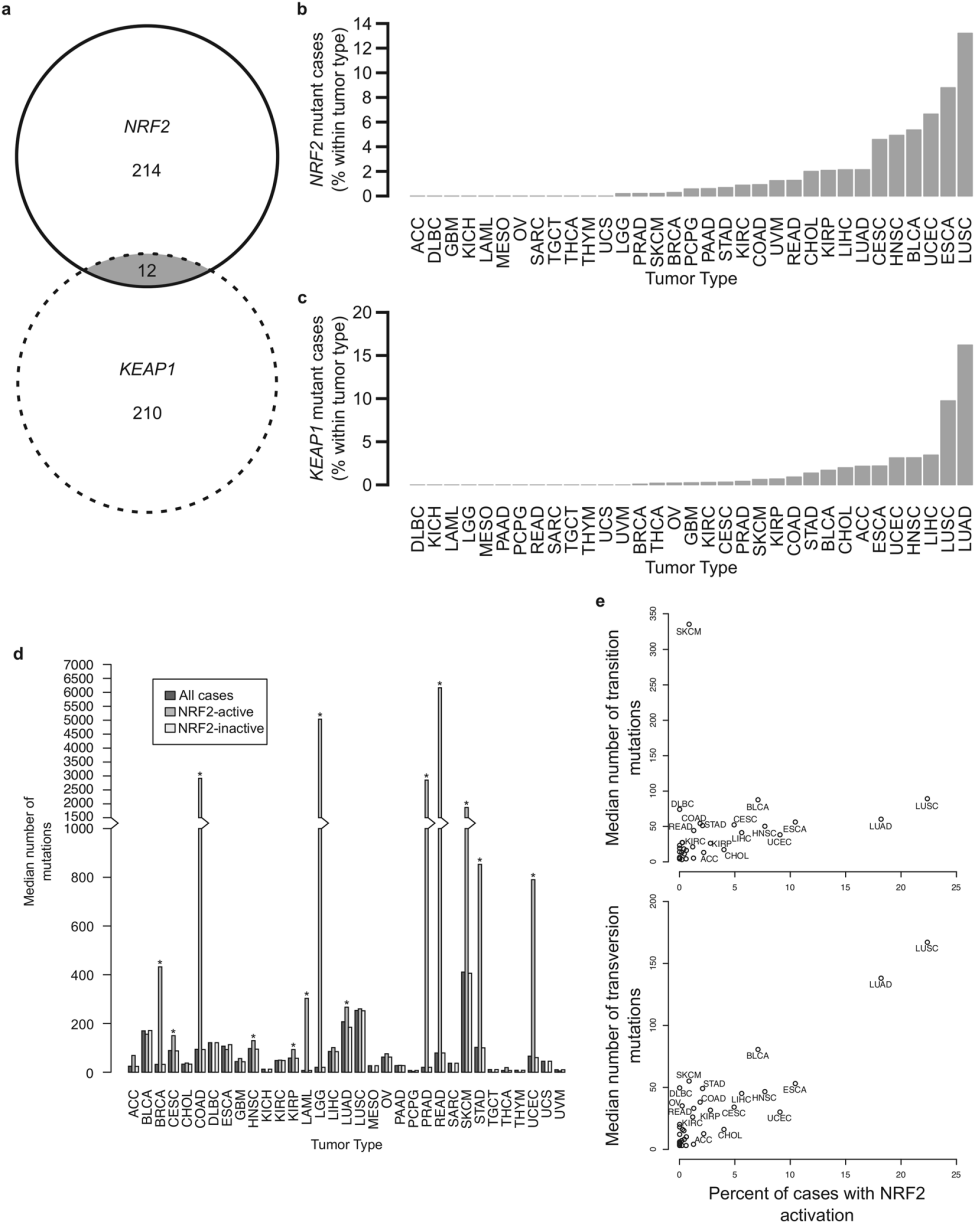
*NRF2* and *KEAP1* mutations are overrepresented in tumors associated with carcinogen exposure. (**a**) Number of cases identified with *NRF2* mutation, *KEAP1* mutation, or both. (**b**) Percentage of cases within tumor types harboring non-synonymous *NRF2* mutations. (**c**) Percentage of cases within tumor types harboring non-synonymous *KEAP1* mutations. (**d**) Median number of mutations per case by tumor type. Median number of mutations in NRF2-active cases was significantly higher than that in NRF2-inactive cases for the tumor types indicated (*indicates p < 0.05). (**e**) Median number of transversion mutations positively correlates with percentage of cases harboring NRF2 activation (p < 0.05), but median number of transition mutations does not (p > 0.05).

There were a total of 226 cases of tumors with somatic *NRF2* and 222 cases with somatic *KEAP1* mutations. Of these, 12 cases harbored both *NRF2* and *KEAP1* mutations. Random sampling showed that the overlapped 12 cases could have happened by random chance alone (Fig. 2a). Consistent with the literature, certain tumor subtypes show higher representation of either somatic *NRF2* or *KEAP1* mutations. Somatic *NRF2* mutations were most frequently found in lung squamous cell carcinoma (LUSC), followed by esophageal carcinoma (ESCA) and uterine corpus endometrial carcinoma (UCEC) cancers (Fig. 2b). Somatic *KEAP1* mutations were most frequently found in lung adenocarcinoma (LUAD), followed by LUSC and liver hepatocellular carcinoma (LIHC) (Fig. 2c). There is also a strong overlap among tumor types with somatic *NRF2* or *KEAP1* mutations, and many of these tumor types are associated with exposure to xenobiotics^28^, suggesting carcinogen exposure as a selection pressure that selects for cells with sustained NRF2 activation phenotype. Many tumor types with NRF2 activation (either *NRF2* or *KEAP1* non-synonymous mutation) show increased frequency of mutations (Fig. 2d). Moreover, across tumor type, there is a significant correlation between the frequency of NRF2 activation and transversion mutation events (correlation = 0.89, p-value = 7 × 10^−12^), but not to transition mutation events (correlation = 0.21, p-value = 0.23) (Fig. 2e). These correlations hold true even when the analyses were performed on either *NRF2* or *KEAP1* mutations alone (*NRF2-transversion* correlation = 0.69, p = 9.34 × 10^−6^; *KEAP1-transversion* correlation = 0.83, p = 1.77 × 10^−9^; *NRF2-transition* correlation = 0.20, p = 0.27; *KEAP1-transition* correlation = 0.17, p = 0.34) (Supplementary Fig. S1). This is indicative of carcinogen exposure, as transversion mutations are associated with exposure to alkylating agents. The significant correlation between NRF2 activation and carcinogen-associated transversion mutations is in agreement with the known correlation between *NRF2* and *KEAP1* mutations and cancer cases from smokers^8^. Consistently, the NRF2 target gene, aldose ketose reductase family 1 member B10 (*AKR1B10*), was also reported as a reliable biomarker for smoking in lung cancers, and its expression is a good surrogate for NRF2 activation in lung cancer^29,30^. More recently, Orrù and co-workers demonstrated that diethylnitrosamine, an alkylating agent, induced a high frequency of *NRF2* gain-of-function mutations in a rat liver carcinogenesis model. The mutations were found in the preneoplastic lesions. Moreover, they showed that *NRF2* gain-of-function mutations were critical for the onset of hepatocellular carcinoma in the model^31^.

### R34 is the most frequently mutated amino acids in NRF2

**Figure 3:**
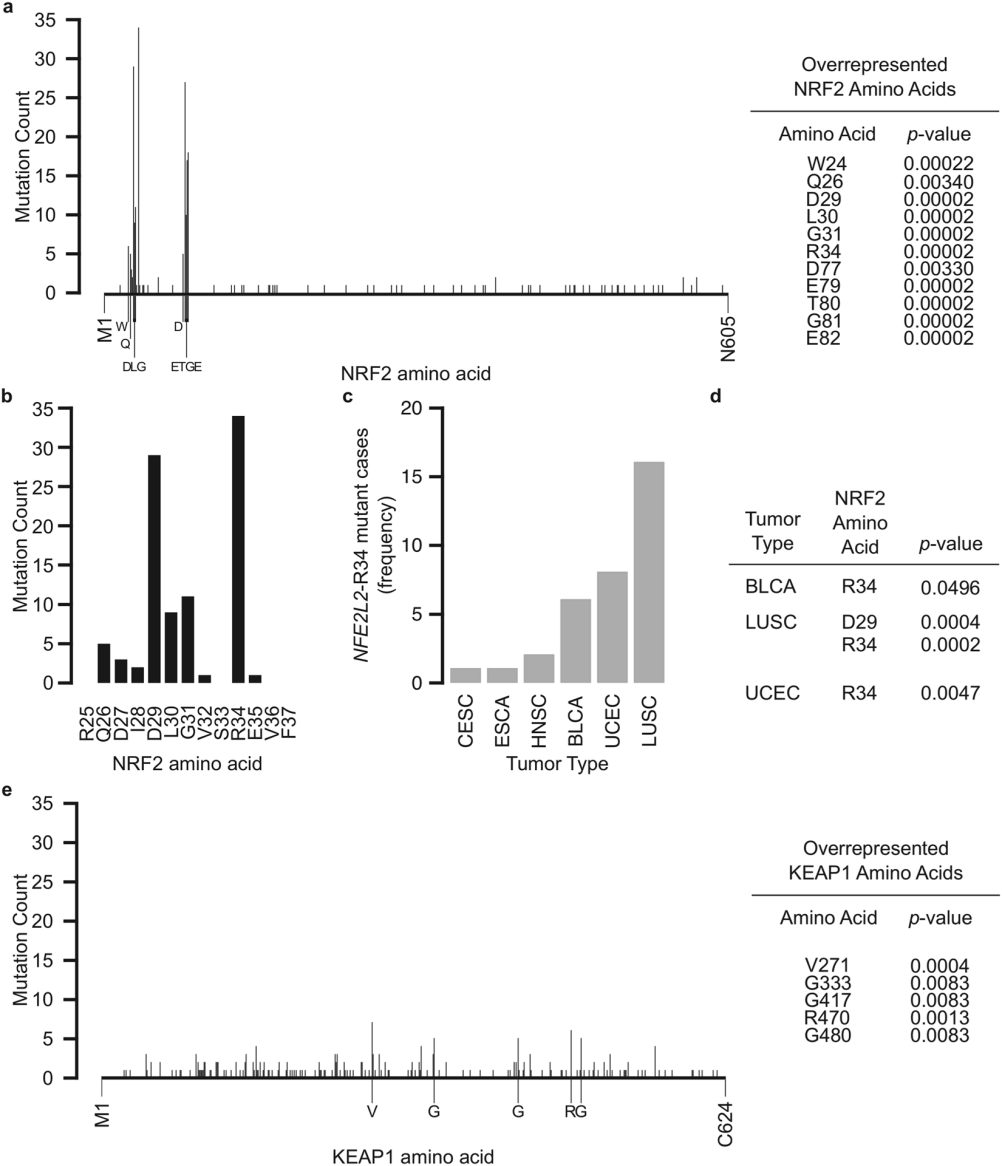
*NRF2* and *KEAP1* are preferentially mutated at positions including and beyond *NRF2*-DLG and *NRF2*-ETGE motifs. (**a**) Number of nonsynonymous mutations at each NRF2 amino acid position from M1 to N605. Labeled amino acids were significantly enriched (p < 0.05). (**b**) Number of nonsynymous mutations at each NRF2 amino acid position along the DLG motif, shown from R25 to F37. R34 was the most frequently mutated NRF2 amino acid in the entire protein within this dataset. (**c**) Frequency of R34 mutant cases by tumor type. (**d**) R34 mutation is significantly enriched (p < 0.05) in specific tumor types. (**e**) Number of nonsynonymous mutations at each KEAP1 amino acid position from M1 to C624. Labeled amino acids were significantly enriched (p < 0.05).

NRF2 protein consists of 605 amino acids, and mutations were mainly found within the NEH2 domain, where the ETGE and DLG motifs are located (Fig. 3a). Several mutations outside of the DLG and ETGE motifs, including W24, Q26, R34, and D77, were found to be significantly overrepresented. Upon closer inspection, we found that R34 is the most frequently mutated residue of *NRF2* (Fig. 3b), and accounted for 14.2% of all *NRF2* mutations. Tumor types distribution analysis showed R34 mutations were found in tumors of bladder, cervical, esophageal, head and neck, lung, and uterine origins (Fig. 3c). The R34 was also the only amino acid position to be preferentially enriched within several tumor types, including bladder, lung squamous cell, and uterine cancers (Fig. 3d).

Contrary to *NRF2* mutations, *KEAP1* mutations spread across the length of the entire protein (Fig. 3e). Intriguingly, five *KEAP1* amino acids were preferentially mutated (Fig. 3e). Positions where *NRF2* and *KEAP1* mutations were overrepresented were significantly enriched for transversion mutations (Supplemental Fig. S2).

### RNA sequencing data revealed amino acid changes that activate NRF2

**Figure 4:**
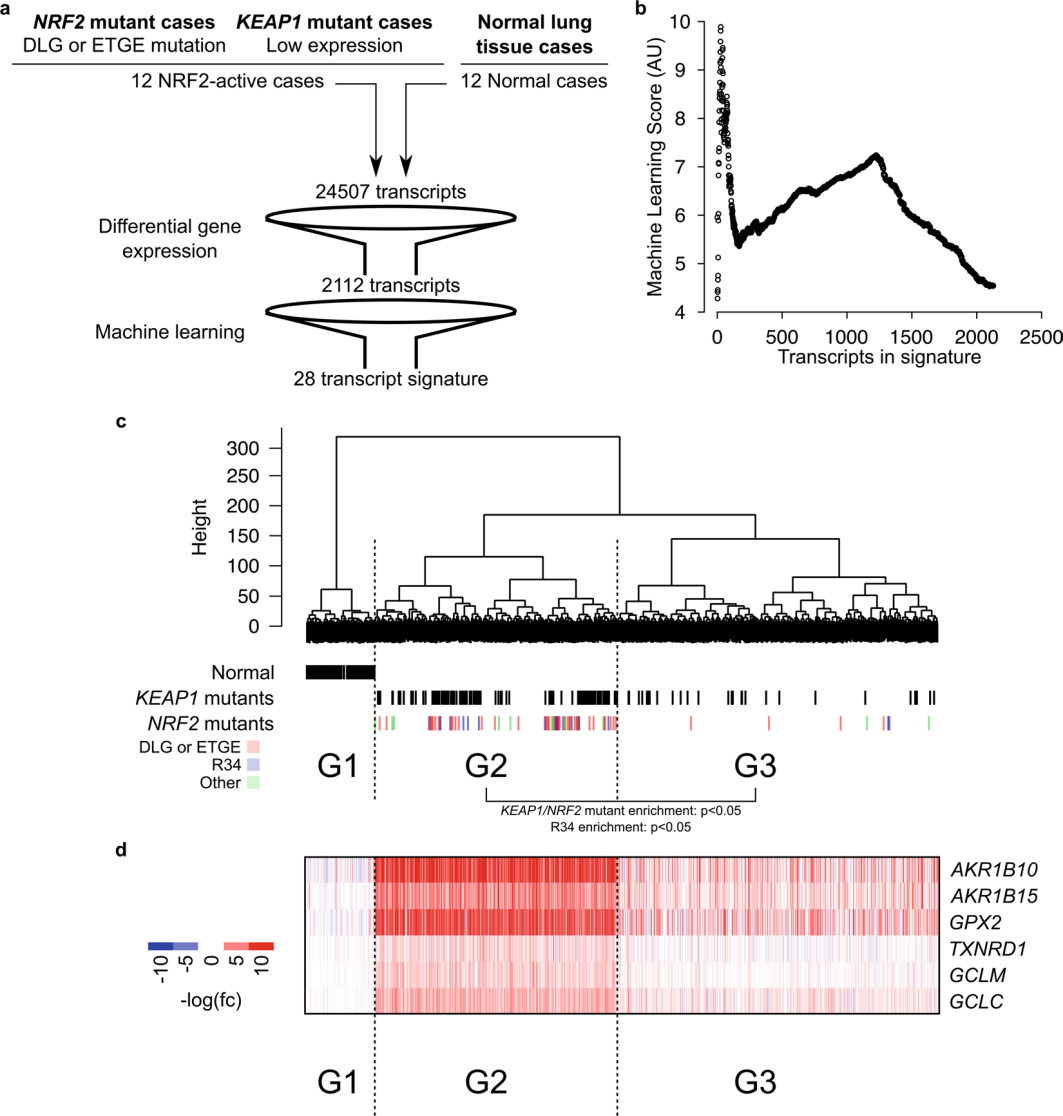
RNA sequencing reveals NRF2-activating mutation. (**a**) Schematic showing workflow for identifying an NRF2-activation gene signature. (**b**) Machine Learning Scores for sequential addition of genes identified 28 genes as the highest-scoring geneset. (**c**) Hierarchical clustering analysis of RNA sequencing data of all lung tumor (LUSC and LUAD) cases using Ward’s minimum variance method with the 28 gene signature. G1 was designated as the normal tissue cases, G2 as the NRF2-active group, and G3 as the NRF2-inactive group. Individual cases designated as normal, *KEAP1*-mutant, or *NRF2* mutant are indicated with vertical lines below their clustered position. *KEAP1* and *NRF2* mutants were enriched in G2 relative to G3 (p < 0.05). R34 mutants were enriched in G2 relative to G3 (p < 0.05). (**d**) Heatmap showing coordinated increased expression of canonical NRF2 target genes in the G2 lung tumor cases.

Having catalogued somatic *NRF2* mutations, we sought to determine whether those mutations have a functional impact. Given that NRF2 activation promotes the transcription of its target genes, we evaluated NRF2 activation using the corresponding gene expression profiles. We focused our analysis on lung cancer, both LUSC and LUAD, since these are the tumor types with the most *NRF2* and *KEAP1* mutant cases. To perform the evaluation in an unbiased manner, we utilized a machine learning approach to construct an NRF2 activation signature (Fig. 4a). A subset of RNASeq data from 12 NRF2 activated tumor and 12 normal control cases were set aside as a training set. These 12 NRF2 activated tumor cases consisted of 6 cases (3 LUSC and 3 LUAD) with *NRF2* mutations and 6 cases (3 LUSC and 3 LUAD) with *KEAP1* mutations. The 6 *NRF2* mutant cases were chosen based on mutation at the DLG or ETGE motifs, which are known to activate NRF2, while the 6 *KEAP1* mutant cases were chosen based on low *KEAP1* expression levels. We performed differential gene expression analysis to identify genes that are differentially regulated between tumor and normal tissues. This analysis filtered the number of transcripts from 24,507 to 2,112. Since the training set consisted of two tumor types (LUSC and LUAD), we devised a scoring algorithm to remove the tumor type biased (described in Methods section). Using this scoring system, genes were ranked and the 28 top scoring genes were chosen as the NRF2 activation signature (Supplemental Table S1). We have chosen to use 28 top scoring genes because it is the number of genes that gives the best stratification of the cases in the training set (Fig. 4b). Using this signature, we evaluated NRF2 activity in 995 lung cancer cases. We identified that 423 cases were deemed to have constitutive NRF2 activation (Fig. 4c). Accordingly, these cases showed coordinated upregulation of classical NRF2 target genes, *AKR1B10*, *AKR1B15*, *GPX2*, *TXNRD1*, *GCLM*, and *GCLC* (Fig. 4d). Furthermore, out of these 423 cases, 165 cases harbored either *NRF2* or *KEAP1* mutation. The analyses also indicated that the most frequently occurred R34 mutant is an activating mutation. The complete catalogue of lung NRF2 mutations with transcriptomic data, their frequency of occurrence, and the inferred impact on activity are summarized in Supplemental Table S2. A handful of unique mutations appeared in both the NRF2-activated and NRF2-inactivated grouping; this may be caused by tumor heterogeneity between tissue sections used for exome and RNA sequencing.

To evaluate the functional impact of *NRF2* and *KEAP1* mutations identified in the activating signature, we utilized a luciferase reporter construct under the control of the antioxidant response element (ARE) enhancer. NRF2 binds to ARE sequence to drive expression of a luciferase reporter that serves as an indicator for NRF2-mediated transcriptional activation. We developed NRF2 expression constructs for the non-DLG and non-ETGE *NRF2* mutants found in the activated signature, as well as the R34L *NRF2* mutant. All mutants enhanced luciferase activity to levels similar as wildtype NRF2, indicating none of the mutants were deleterious to NRF2 activity (Supplementary Fig. S3). As expected, KEAP1 expression ablated wild type NRF2 luciferase activity to <10% of its original level. However, KEAP1 introduction reduced luciferase activation by NRF2-R34G, -R34P, -R34L, and -R34Q to only 60%; similarly, W24C, Q26R, Q26L, Q26P, D77G, and D77Y retained significant activity after KEAP1 expression. Activity of some mutants found in the signature (H107R, M235I, F289L, and L370V) was still repressed by KEAP1, indicating a possible secondary mechanism for NRF2 activation may be present in those tumors (Supplementary Fig. S3). We also evaluated several of the *KEAP1* mutations found in the activating signature, focusing on those mutation positions that were overrepresented (Fig. 3e). All of the *KEAP1* mutations evaluated were unable to repress NRF2-mediated luciferase activity as effectively as wild type KEAP1 (Supplementary Fig. S3). This demonstrates that many of the *NRF2* and *KEAP1* mutations identified in the NRF2-activating signature modulate NRF2 activity.

### Mutational bias at R34 position of NRF2

**Figure 5:**
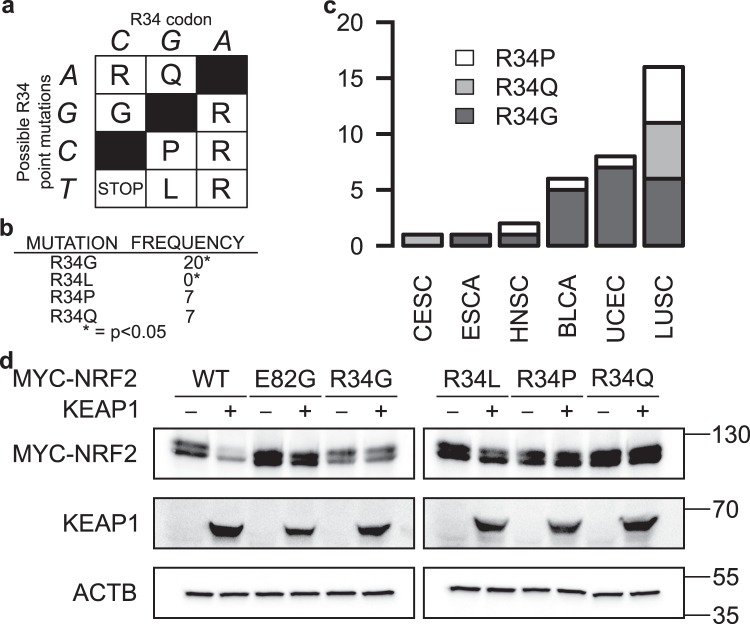
R34 mutation is biased against R34L. (**a**) Genetic code table of possible mutations to R34 codon. (**b**) R34G was significantly enriched amongst the 34 R34 mutations identified, while R34L was absent in the TCGA dataset. (**c**) Distribution of the different variants of R34 mutations across tumor types. (**d**) Western blots of HEK293 cells co-transfected with MYC-tagged wildtype (WT) or mutant NRF2 and either KEAP1 (+) or empty vector control (−). KEAP1 overexpression cannot decrease NRF2-R34 mutants’ protein levels, except NRF2-R34L. ACTB was used as a loading control. MYC-NRF2 and KEAP1 were resolved on the same 7% SDS-PAGE gels for corresponding sets of samples, while ACTB was resolved on 15% gels. WT, E82G, and R34G were run on one set of gels, while R34L, R34P, and R34Q were run on a separate set of gels. Uncropped blots are available in Supplementary Fig. S5.

Since mutation at the R34 position is the most frequently occurring *NRF2* mutation and is located outside the ETGE and DLG sites known to be essential for KEAP1 binding, we sought to further evaluate mutational changes that are relevant to this position. The R34 of NRF2 is encoded by a CGA codon. Thus, a single point mutation to this codon can result in either R34G, R34Q, R34P, or R34L amino acid change (Fig. 5a). All of these mutants retained NRF2-mediated ARE-luciferase activity (Supplementary Fig. S3). Upon evaluating the TCGA mutation database, we found that R34L was not represented in the 10,364 cases of tumors evaluated in this study, while R34G was significantly overrepresented (Fig. 5b). The R34G mutation could be found in 5 of the 33 evaluated tumor types (Fig. 5c). To empirically evaluate the functional impact of each of these R34 mutants *NRF2*, we co-expressed each of these mutants with KEAP1 and found that all these mutations protect NRF2 against KEAP1-mediated degradation, with R34L showing the least protective effects among them (Fig. 5d, Supplementary Fig. S4).

Fukutomi and co-workers have shown through *in vitro* binding assay between ETGE-deleted NEH2 domain of NRF2 and KEAP1 that R34Q mutation impairs the DLG-KEAP1 interaction^32^. As binding at both DLG and ETGE motifs is required for ubiquitylation of NRF2^33^, we assessed whether R34 mutants impaired KEAP1’s ability to mediate NRF2 ubiquitylation. As expected, mutations to R34 position showed lower ubiquitylation (Supplementary Fig. S4).

### Mutation at R34 stabilizes NRF2 with R34L conferring the least stabilization

**Figure 6:**
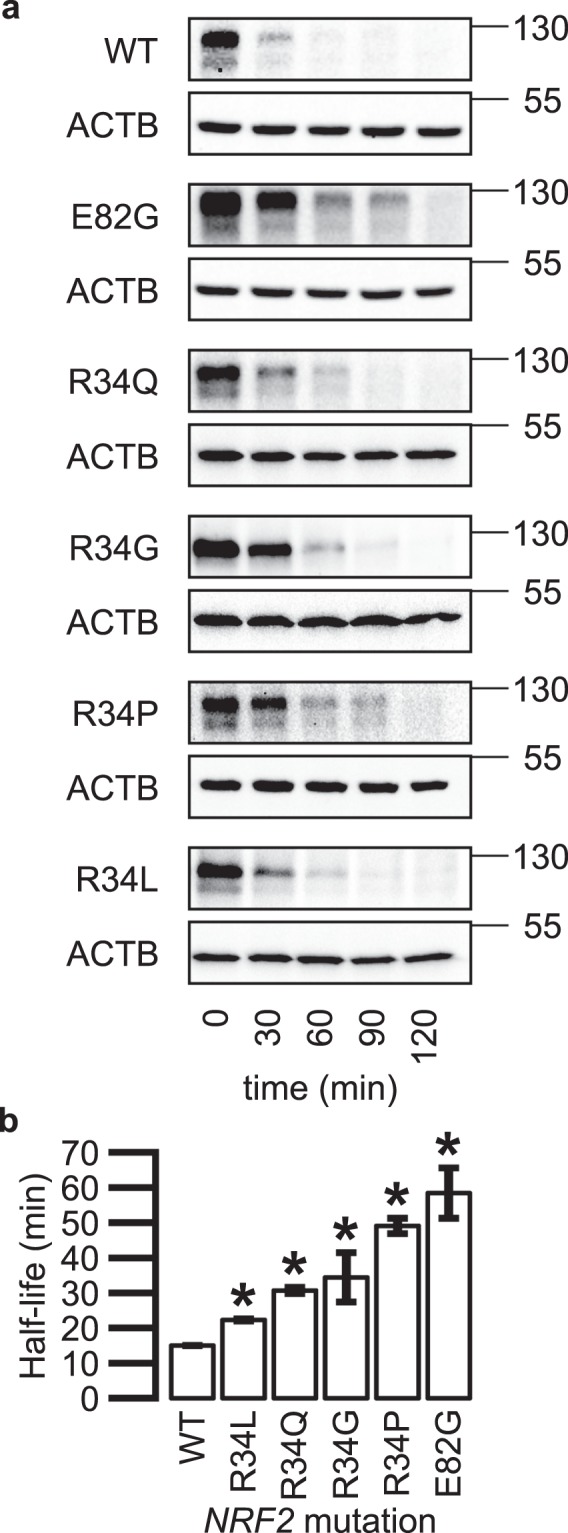
Mutation at R34 extends NRF2 half-life. (**a**) Representative western blot of MYC-tagged wildtype (WT) or mutant NRF2 protein levels decreasing over time following cycloheximide treatment (100 μg/mL). ACTB was used as a loading control. MYC-NRF2 was resolved on 7% SDS-PAGE gels, while ACTB was resolved on 15% gels. (**b**) NRF2 wildtype and mutant protein half-lives calculated from densitometry analyses of 3 biological replicates of cycloheximide chase. *Indicates p < 0.05 relative to WT by 2-tailed t-test and error bars represent ± SD. Uncropped blots are available in Supplementary Fig. S6.

To empirically determine the stability of the different *NRF2* R34 mutants, we performed cycloheximide chase assays to determine the half-life (t_1/2_) of each mutant. The analysis showed that R34L is the least stable mutant (Fig. 6a). Subsequent t_1/2_ estimation showed that the R34L has a t_1/2_ of 22 minutes compared to 15 minutes of wild-type NRF2 (Fig. 6b). The *NRF2*-E82G mutant, which was used as a positive control, was the most stable, with a t_1/2_ of 58 minutes. The t_1/2_ of other *NRF2*-R34 mutants were in between 31 and 49 minutes, with R34L having the shortest half-life. Thus, the absence of R34L in the cases analyzed may indicate that the increased stability conferred by R34L mutation does not pass the transcriptional reprogramming threshold to be positively selected during cancer development and progression.

## Discussion

Activating the NRF2 transcription factor has long been recognized as a means to protect cells against chemical carcinogenesis and environmental insults^34–38^. However, recent studies revealed that many tumors exhibit constitutive NRF2 activation driven by either somatic mutation to *NRF2* itself or to its regulatory genes^11–13^. Importantly, tumors with constitutive NRF2 activation are more aggressive and are more resistant to most treatment modalities, prompting the need to identify tumor cases with *NRF2* mutations for potential patients stratification and to develop NRF2 inhibitors^39^. Given the role of NRF2 in protecting tissues against redox insults, exploiting NRF2 inhibition as a treatment strategy needs to be tumor selective as inhibiting NRF2 in normal cells may lead to increased cellular oxidative damage, increased cytotoxicity of chemotherapeutics, and increased susceptibility to malignant transformation. One strategy to target cancer specific proteins is with inhibitors that target the mutant variant of the protein: drugs such as gefitinib (against mutant EGFR) and vemurafenib (against BRAF V600E) have a good degree of success in cancer management^40,41^. The development of such mutant specific compounds requires that cancer specific mutations happen in a predictable manner. For example, oncogenic BRAF mutations regularly occur at the P-loop and the activation domain (V600 is located within the activation domain)^42^. Thus, drug development efforts can concentrate on developing compounds that target the cancer specific variant, while leaving wild-type protein in normal cells untouched. This study demonstrates that somatic *NRF2* mutations in cancer fulfill this criterion. Specifically, the distribution of somatic *NRF2* mutations are very similar to those sustained by oncogenes whereby the mutation are focal at certain locations. As such, it opens up opportunities to develop mutant specific NRF2 inhibitors, allowing tumor specific NRF2 inhibition while leaving wild type NRF2 in normal tissues to carry out its protective functions. Additionally, the nature of the focal mutations also allow for individualized mutation screening, whereby the mutational hotspots can be amplified with appropriate PCR primers followed by Sanger sequencing. Such screening strategy may be developed into a diagnostic method when mutant specific NRF2 inhibitors become available.

Consistent with previous studies^7,8^, our results also showed that somatic *NRF2* mutations primarily occurred at the ETGE and the DLG motifs, which interfere with KEAP1 binding. Several mutations outside the DLG/ETGE motifs to *NRF2* were also frequently mutated (p < 0.05), including W24, Q25, D77, and R34. While a few isolated mutations to these locations have been reported and shown to mitigate NRF2-KEAP1 interactions^32^, we focused on the R34 mutation and found it to be the most frequently mutated amino acid position and the only position significantly enriched across many tumor types. We identified 34 cases harboring R34 mutations; these are high confidence mutations, as they were called by at least two of the mutation-calling algorithms used, and 30/34 mutations were called by all four algorithms. Upon evaluation of possible R34 mutations, there is a selection bias against R34L mutation. Using ARE-Luciferase reporter assay, we found that R34L mutant could still evade KEAP1-mediated decrease in NRF2 transcriptional activity. Half-life analysis showed that R34L mutation still stabilizes NRF2. However, it is the least stable among the *NRF2* R34 mutants, indicating a potential minimal stability threshold for NRF2 to benefit cancer growth. Although mechanistic underpinning behind the overrepresentation of longer half-life *NRF2* mutants in cancer is still lacking, constitutive/sustained versus intermittent NRF2 activation has been proposed as the distinguishing feature that separate cancer prevention and cancer promotion properties of NRF2 activation^13,43^. Given that *NRF2* R34L has the shortest half-life and is significantly negatively selected for in cancer, future studies into the potential periodicity of *NRF2*-R34L activation may offer clues into the hypothesis behind constitutive versus intermittent NRF2 activation in cancer prevention and promotion.

Apart from somatic *NRF2* mutation, somatic mutations that activate NRF2 can range from direct loss-of-function KEAP1/CUL3 mutations^6,9,10^, to *KEAP1* gene silencing^44^, to somatic mutations that lead to accumulation of oncometabolites^45–47^, or ETGE/ETGE-like motifs containing proteins^48^. Intriguingly, our analysis revealed several *KEAP1* mutations that were significantly enriched; some of these locations have been characterized, including *KEAP1*-G333C which has been shown to not bind NRF2 and subsequently not suppress NRF2-mediated transcription. In contrast, *KEAP1*-R470C mutants have exhibited enhanced NRF2 binding: these “superbinder” mutants were shown to not suppress NRF2-mediated transcription, albeit through an unknown mechanism^49^. Identifying which biochemical class of *KEAP1* mutant those identified in this manuscript fall under could lead to novel insights into NRF2-KEAP1 relationships.

Stratification of the lung cancer cases based on our NRF2 activation identifier revealed a large number of cases appeared to have NRF2 activation yet did not harbor an *NRF2* or *KEAP1* mutation. Thus, mutations to other genes may also contribute to the observed NRF2 activation phenotype. Given the prognostic and potential treatment implications of identifying cases with NRF2 activation, there is a need to identify all NRF2 regulatory genes, which when mutated drive constitutive NRF2 activity. To date, much of what is known is based on piece-meal efforts of identifying one regulatory gene at a time; genome wide systematic identification of the NRF2 regulatome may offer a more powerful way of reanalyzing TCGA and other legacy data to identify the mechanisms by which NRF2 becomes activated in tumors.

Of the genes identified in our NRF2 activation signature, *AKR1B10* was the only bona fide NRF2 target gene. *AKR1B10* can be found in other NRF2-activation gene expression signatures^19,50^, and its overexpression has been associated with lung cancers in particular^51^. Besides *AKR1B10*, one gene in our signature, *NR0B1*, has been utilized in other NRF2-activation signatures^50,52,53^; how NRF2 regulates *NR0B1* expression has not been identified. The other genes identified in our signature have not been found in several other signature we looked at for NRF2 activation^15,19,50,53^. It remains unclear if and how these genes are regulated by NRF2.

Apart from mutation sites and gene expression changes, the overrepresentation of *NRF2* and *KEAP1* mutations in tumor types with known association with carcinogen exposure provides a glimpse into the roles of NRF2 in cancer development and progression. We identified that tumors with *NRF2* or *KEAP1* mutations show higher frequencies of transversion mutations. Many carcinogen-associated Michael acceptors, such as acrolein in cigarette smoke or quinone metabolites of polyaromatic hydrocarbons found in exhaust fumes, are known to cause both transversion mutations and NRF2 activation^54–60^. Given the role of NRF2 in protecting cells against environmental insults, exposure to environmental carcinogens like cigarette smoke may exert a selection pressure for cells with constitutive NRF2 activation. Thus, NRF2 activation may allow cells to endure the insults from mutagens and carcinogens, and survive to undergo malignant transformation. Indeed, recent work by Orrù and co-workers showed that *NRF2* activating mutations were acquired during early carcinogenesis in a rat carcinogenesis model using diethylnitrosamine (an alkylating agent) as the carcinogen. They also showed that NRF2 activation was necessary for expansion of initiated cells^31^. However, we cannot rule out the possibility that NRF2-activating gene mutations contribute toward cancer progression rather than development.

## Conclusions

This study provides an overview of NRF2 mutations in cancer in one of the largest curated datasets presented. We identified four *NRF2* mutation hotspots that fall outside the well-established DLG and ETGE motifs, and five *KEAP1* mutation hotspots. Of all *NRF2* mutant hotspots, R34 was the most frequently mutated. Functional and transcript analyses revealed R34 mutation prevents KEAP1-mediated NRF2 degradation, stabilizes the protein, and leads to NRF2 activation. Future computational approaches may focus on the development of a web application that integrates existing knowledge on NRF2 signaling, allowing easy indexing and consolidation of knowledge that may guide prediction of cellular changes following specific alterations to the NRF2 signaling pathway.

## Methods

### TCGA mutation data

Somatic mutations and RNASeq data were downloaded from The Cancer Genome Atlas (TCGA) consortium (http://cancergenome.nih.gov/) on March 2, 2017. Somatic mutation data from 33 tumor types identified using 4 different somatic mutation-calling algorithms (MuSE, MuTect2, SomaticSniper, and VarScan2) were utilized in the analyses.

### Statistical analyses

All statistical analyses were performed in R statistical environment^61^. Correlations between mutation type and frequency of NRF2 activation, *KEAP1* mutation, or *NRF2* mutation were performed using Pearson’s product-moment correlation test. Correlation p-values were calculated according to Fisher’s transformation. P-values for mutation enrichment/overrepresentation were estimated by Monte Carlo simulation with the estimated p-values calculated as *P(b)* = *(b* + *1)/(m* + *1)*, where *b* is the number of permutations with enrichment frequency greater than or equal to that observed in the TCGA data and *m* is the number of random permutations, which is 5 × 10^6^ for each estimated p-value. For efficiency, permutations were performed using an in-house C++ implemented R function utilizing the Rcpp package^62^. All p-values were adjusted for multiple testing according to the method proposed by Benjamini and Hotchberg. An estimated p-value of <0.05 is deemed significant.

### RNA sequencing analysis

Raw RNA sequencing count data for non-small cell lung cancers (LUAD and LUSC) were used in RNAseq analyses. Differential gene expression between tumor and normal lung tissues was assessed using the DESeq 2 package^63^. Genes with an adjusted p-value of <0.05 and at least 2.5 log2 fold change were deemed significant and utilized for downstream analysis.

Variance stabilized transformed relative expression levels were used to develop an NRF2 activation signature. Twelve tumor and 12 normal tissues were set aside as a training set. The 12 tumors in the training set consisted of 6 cases with either *NRF2*-DLG or -ETGE motif mutations (3 LUAD and 3 LUSC cases) and 6 cases with low relative KEAP1 expression amongst *KEAP1* mutant cases (3 LUAD and 3 LUSC cases), while the 12 normal tissues were consisted of 6 cases of LUSC and 6 cases of LUAD normal tissues. To minimize tumor type effects (LUSC vs LUAD), the absolute difference in relative gene expression level between LUAD and LUSC, |d_r_| were calculated and ranked in increasing order so that genes with the lowest |d_r_| (genes with the least inter tumor type difference will be on the top of the list). Next, sequential hierarchical clustering that progressively adds one gene from the list (starting with the top two genes) into a developing signature per iteration was performed. With each iteration, a machine learning score (MLS) was calculated whereby *MLS* = *L*_*Inter*_/*L*_*Intra*_ where *L*_*Inter*_ is the intergroup Euclidean distance (between normal cases and tumor cases), and *L*_*Intra*_ represents the intragroup Euclidean distance (distance between different individual cases within the same hierarchical cluster). From this sequential clustering analysis, we found that the top 28 genes from the list gave the best distance between the NRF2 activated tumor cases from the NRF2 low normal cases. This 28 genes signature was then used in the testing set, which consisted of all lung cancer cases to test its ability to stratify samples based on NRF2 activation.

### Cell culture conditions

HEK293 cells were obtained from American Type Culture Collection (ATCC) (Manassas, VA). HEK293 cells were cultured in Dulbecco’s modified Eagle’s medium with high glucose (4.5 g/L) (DMEM) and 10% fetal bovine serum (FBS). All FBS was heat inactivated at 56 °C for 30 minutes. Cells were cultured at 37 °C in atmospheric air enriched with 5% CO_2_. For both sustained culture and experiments, HEK293 cells were cultured on flasks and dishes coated with poly-D-lysine.

### Plasmid generation and ectopic gene expression

MYC3-NRF2 (Addgene #21555) and HA2-KEAP1 (Addgene #21556) were obtained from Addgene (Cambridge, MA) following their characterization^64^. HA-Ubiquitin was a gift from Edward Yeh (Addgene #18712)^65^. Site-directed mutagenesis was carried out on MYC3-NRF2 to generate NRF2 mutations. The KEAP1 open reading frame was isolated from HA2-KEAP1 and cloned into pCDNA3.1(+) to generate PCDNA3-KEAP1. Site-directed mutagenesis was carried out on PCDNA3-KEAP1 to generate KEAP1 mutations.

To evaluate the role of KEAP1 in mediating the degradation of different NRF2 mutants, HEK293 cells were transfected with the different NRF2 mutant plasmids and either PCDNA3-KEAP1 or empty PCDNA3.1(+) using Attractene transfection reagent (Qiagen, Valencia, CA). 24 hours post-transfection, cells were prepared for immunoblot analysis. Three biological replicates were used to quantify relative band intensities with ImageJ software.

### ARE-Luciferase assays

Cells were transfected with pGL4.37[*luc2P*/ARE/Hygro] (Promega, Madison, WI), pRL *Renilla* Luciferase (Promega), and indicated empty vectors, NRF2 expression vectors, and KEAP1 expression vectors. 48 hours after transfection with indicated plasmids, cells were assayed for luciferase activity using dual-luciferase reporter assay system (Promega).

### Immunoblotting

Primary antibodies used in this study were raised against β-actin (ACTB) (1:10,000 in milk, Sigma A1978, St. Louis, MO), HA (1:1000 in milk, Cell Signaling, Danvers, MA) MYC (1:1000 in milk, Cell Signaling 2276), and KEAP1 (1:1000 in milk, Cell Signaling 4678).

### Immunoprecipitation

Cells were transfected with indicated plasmids for 48 hours. For ubiquitylation analyses, cells were treated with 10 μM MG132 for four hours prior to harvesting. Cells were harvested in radioimmunoprecipitation buffer and immunoprecipitated overnight at 4 °C.

### Cycloheximide chase assay

HEK293 cells were transfected with indicated plasmids. 48 hours post-transfection, cells were washed with phosphate-buffered saline (PBS) and 100 μg/mL cycloheximide in serum-free DMEM was added to all plates; plates were placed back in 37 °C incubator. After five minutes, cells were lysed in 1X Laemmli sample buffer as a zero timepoint. Lysates were then collected, boiled, and frozen for later immunoblot analysis. Subsequent plates were similarly harvested every 30 minutes for the duration of the chase assay.

Following immunoblotting, three biological replicates of blots were quantified using ImageJ software with ACTB as a loading control. Half-life was calculated according to the exponential decay equation *N*(*t*) = *N*_0_*e*^−*λt*^ where *N(t)* is the quantity at time *t* with *N*_*0*_ = *N(0)* and λ is the rate constant such that $half-life\,({t}_{1/2})=\frac{\mathrm{ln}\,(2)}{\lambda }$. Student’s *t*-test was used to evaluate significance between WT and mutant NRF2 half-lives, with p < 0.05 deemed significant.

## Electronic supplementary material

Supplementary Figures

Supplemental Table S1

Supplemental Table S2

## Data Availability

The datasets analyzed in this manuscript were generated by the TCGA Research Network and are freely available from The Cancer Genome Atlas (TCGA) consortium (http://cancergenome.nih.gov/). Only de-identified, publicly available data were downloaded and used for this study. Patients’ consent and institutional review board approval for the collection of the original data were done by TCGA. All methods were carried out in accordance with relevant guidelines and regulations.
