# Supplementary material for: A catalogue of somatic NRF2 gain-of-function mutations in cancer
Source: Sci Rep. 2018 Aug 27;8:12846. doi: 10.1038/s41598-018-31281-0 (PMC6110754; doi:10.1038/s41598-018-31281-0)
Supplement: Supplementary file 1 — Supplementary Figures [file 41598_2018_31281_MOESM1_ESM.pdf]

Supplementary Figures S1 – S7.

In support of:

A catalogue of somatic NRF2 gain-of-function mutations in cancer

Michael John Kerins, Aikseng Ooi*

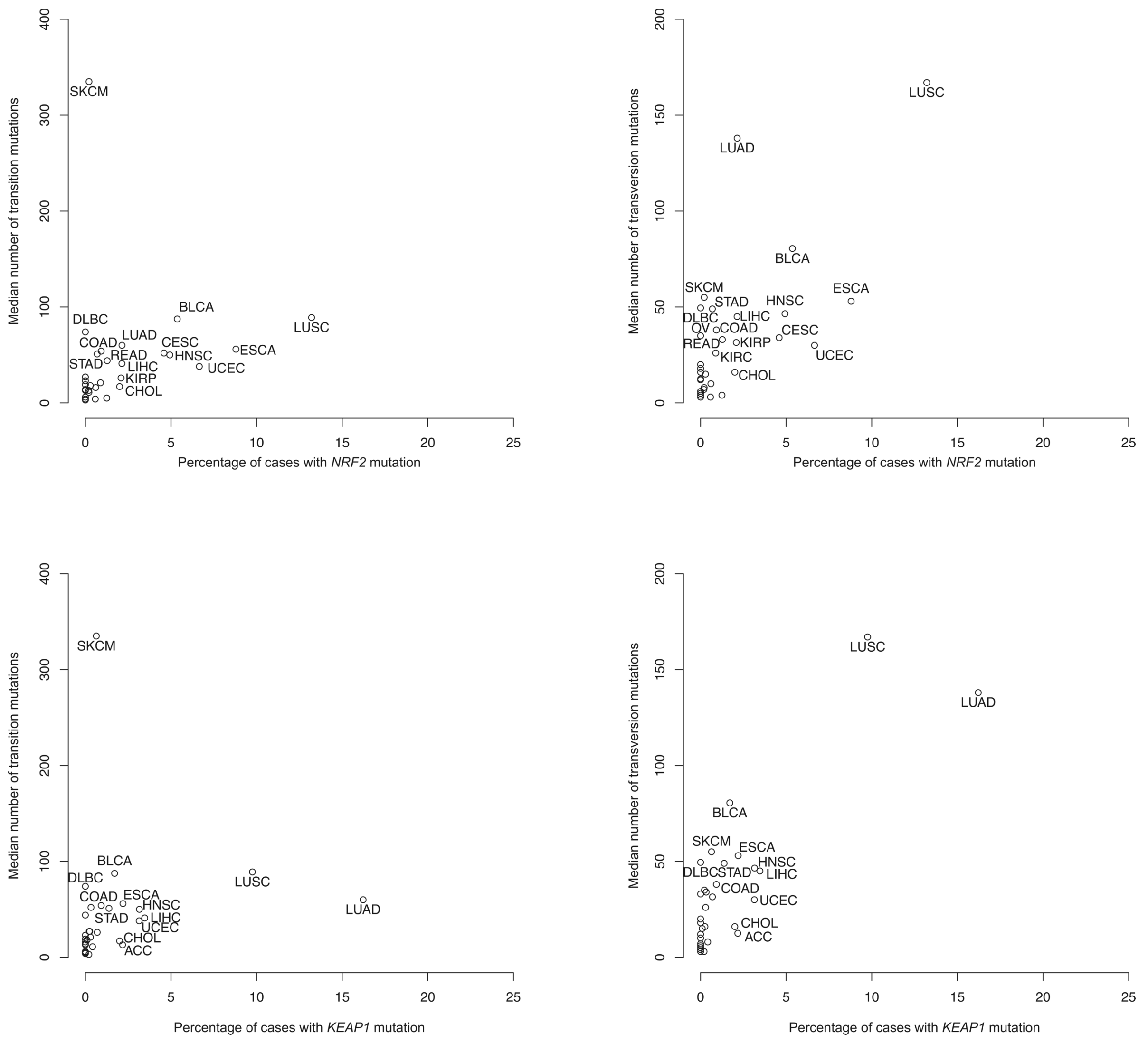

Supplementary Figure S1. Median number of transversion mutations within tumor type positively correlates with percentage of cases harboring *KEAP1* (correlation = 0.83, p = 1.77x10^{-9}) or *NRF2* (correlation = 0.69 , p = 9.34x10^{-6}) mutations within tumor type. Median number of transition mutations within tumor type does not correlate with percentage of cases harboring *KEAP1* (correlation = 0.17, p = 0.34) or *NRF2* (correlation = 0.20, p = 0.27) mutations within tumor type. Correlations were performed using Pearson's product-moment correlation test. Correlation p-values were calculated according to Fisher's transformation

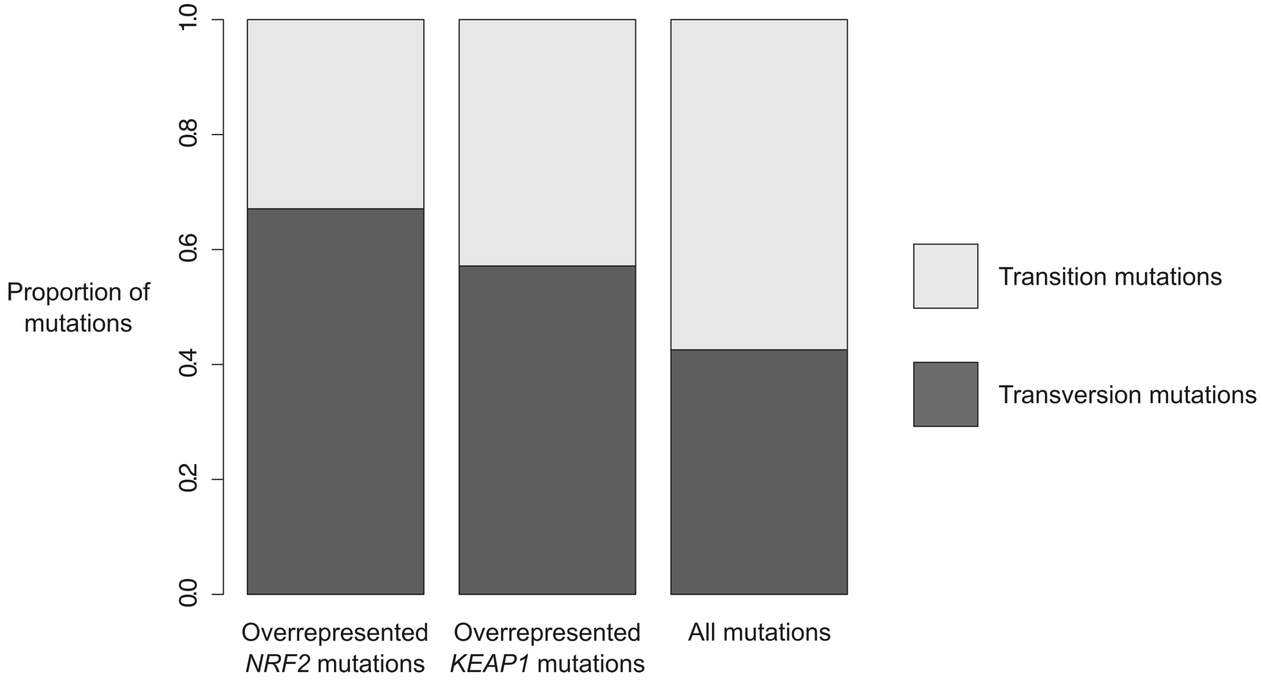

Supplementary Figure S2. Transversion mutations were enriched at overrepresented positions for *NRF2* ($p = 5.767 \times 10^{-15}$) and *KEAP1* ($p = 0.041$) relative to all mutations identified. Enrichment significance was calculated by binomial test.

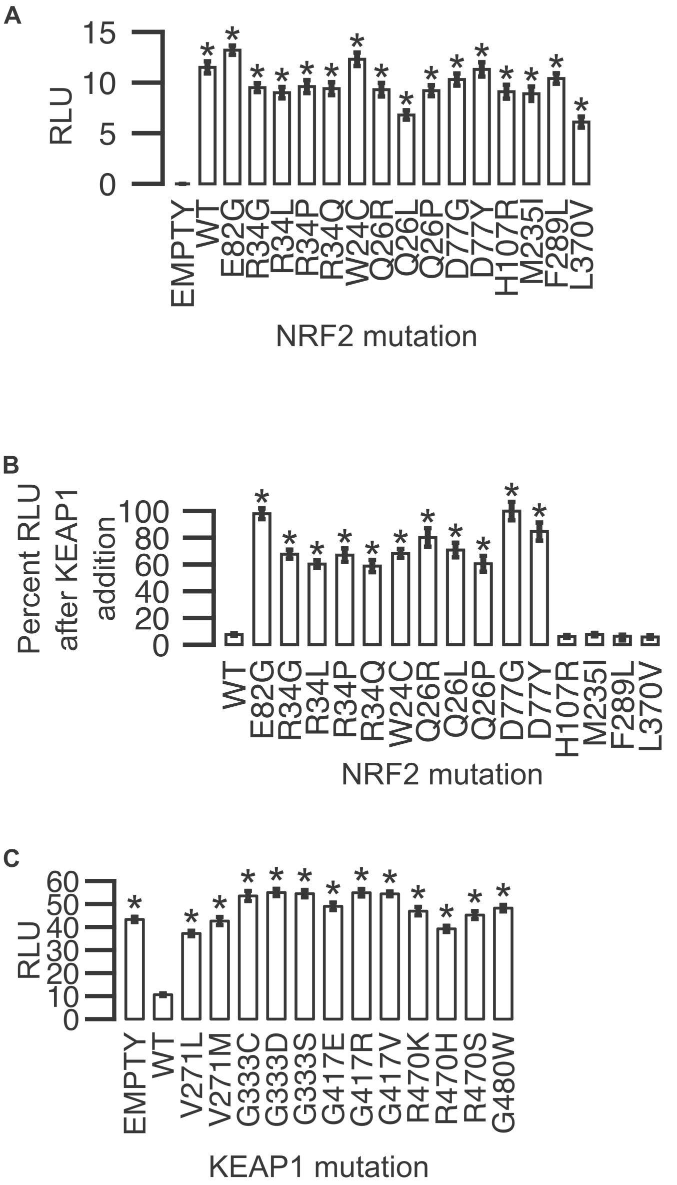

Supplementary Figure S3. The overrepresented *NRF2* and *KEAP1* mutations converge at NRF2 activation. **(A)** Ectopic expression of non-DLG and non-ETGE NRF2 mutants (including to R34 mutants) increased ARE luciferase activity (relative light units, RLU) relative to empty vector, indicating that those mutations do not affect the transcriptional activity of NRF2. **(B)** Percentage of luciferase activity remained when each of those mutants was co-expressed with wild type KEAP1. All NRF2 mutants retained high activity in the presence of KEAP1 except H107R, M235I, F289L, and L370V. * indicates $p<0.05$ by ANOVA and post-hoc Tukey's test relative to EMPTY vector **(A)** or WT NRF2 **(B)**. E82G NRF2 mutant was used as a positive control. **(C)** Wild type NRF2 was co-expressed with KEAP1 mutants, with wild type KEAP1 and EMPTY (wild type NRF2 alone) used as positive and negative controls respectively. Ectopic expression of all mutants did not reduce NRF2-mediated transcription compared to that of wildtype KEAP1. * indicates $p<0.05$ by ANOVA and post-hoc Tukey's test relative to EMPTY vector.

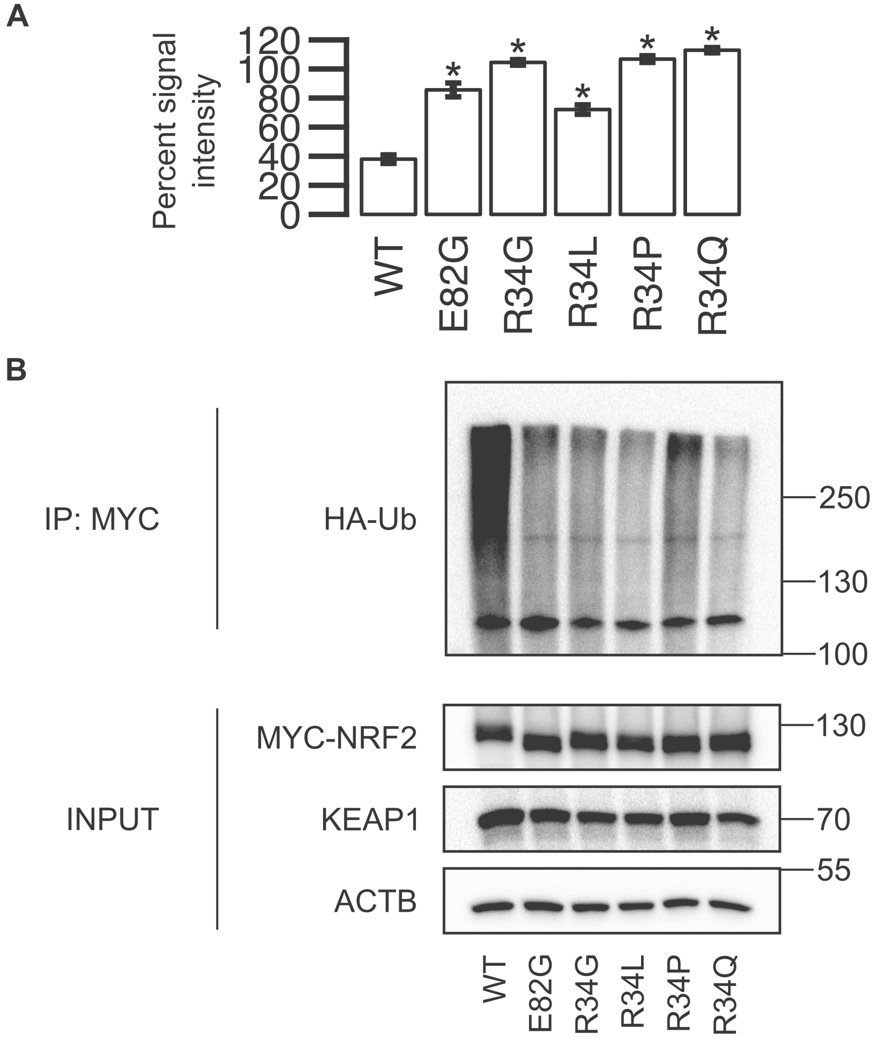

Supplementary Figure S4. (A) Densitometry analysis of 3 biological replicates of results shown in Figure 5D. Percent of MYC-NRF2 western blot signal intensity after KEAP1 addition. Error bar represents SEM. * indicates $p<0.05$ by ANOVA and post-hoc Tukey's relative to WT. **(B)** NRF2-R34 mutants are more resistant to ubiquitylation compared to WT NRF2. Uncropped blots are available in Supplementary Figure S7.

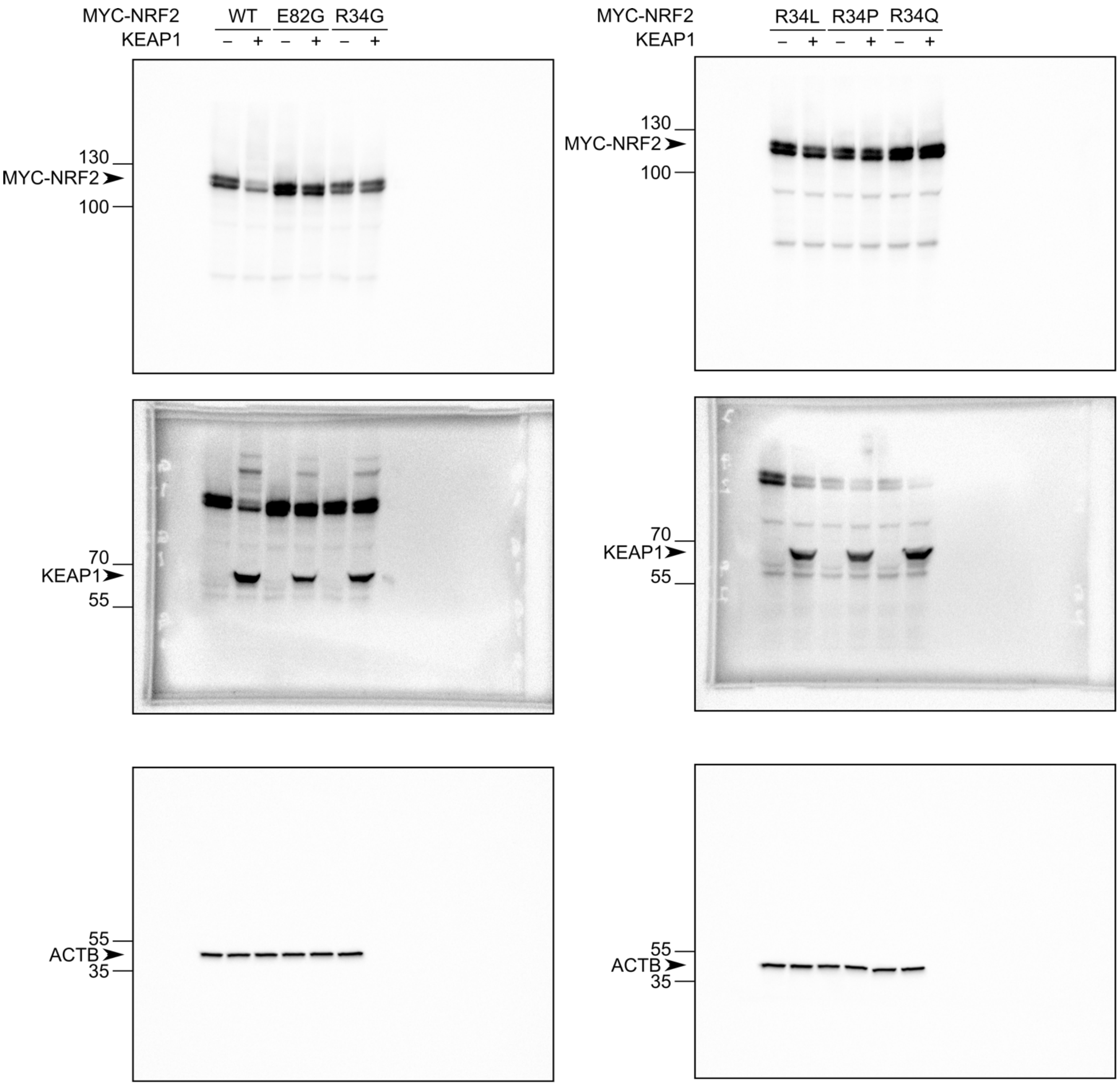

Supplementary Figure S5. Uncropped western blot images associated with Figure 5.

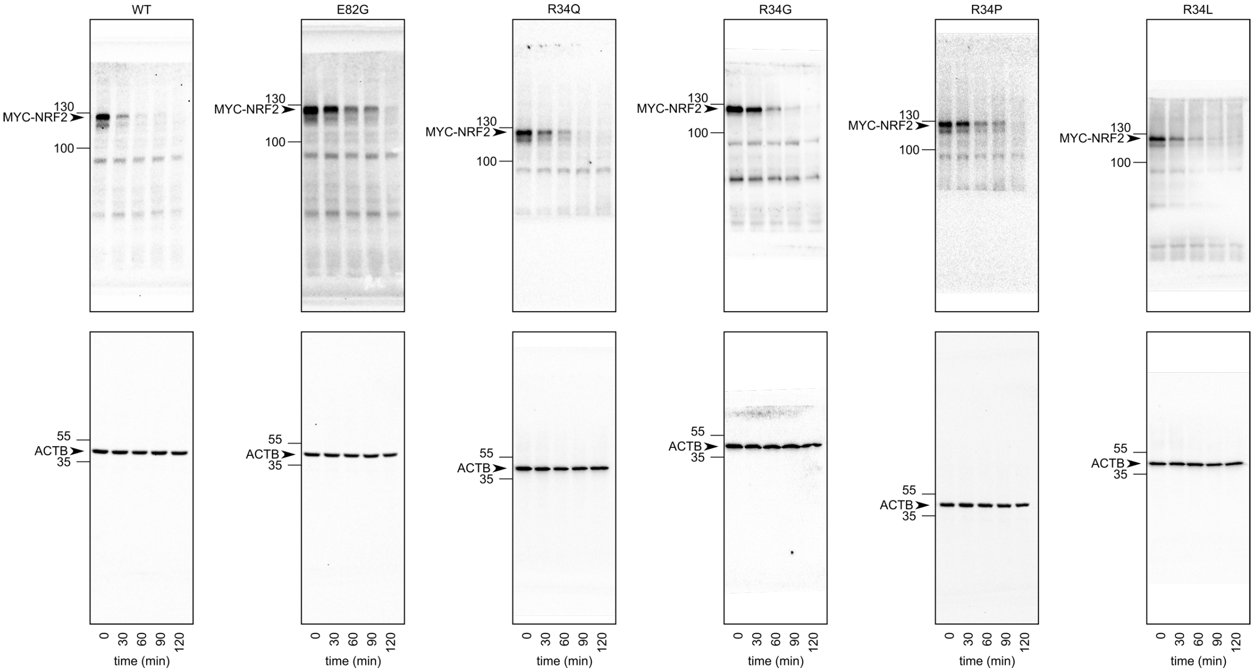

Supplementary Figure S6. Uncropped western blot images associated with Figure 6.

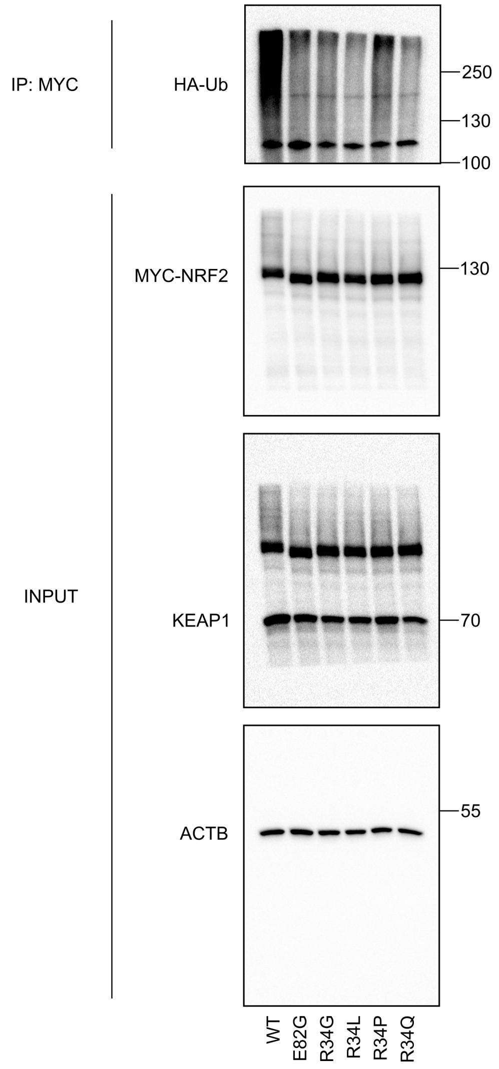

Supplementary Figure S7. Uncropped western blot images associated with Supplementary Figure S4.
